# Supplementary material for: Modeling glioblastoma heterogeneity as a dynamic network of cell states
Source: Mol Syst Biol. 2021 Sep 16;17(9):e10105. doi: 10.15252/msb.202010105 (PMC8444284; doi:10.15252/msb.202010105)
Supplement: Supplementary file 6 — Source Data for Figure 5 [file MSB-17-e10105-s004.zip › Figure5A_sourcedata/GSEA_3017/nosigdb_stateD.GseaPreranked.1622795010397/DOROSZKO_U3054MG_CLUSTER_4.html]

Details for gene set DOROSZKO\_U3054MG\_CLUSTER\_4[GSEA]

|  || Dataset | state23017 |
| Phenotype | NoPhenotypeAvailable |
| Upregulated in class | na\_neg |
| GeneSet | DOROSZKO\_U3054MG\_CLUSTER\_4 |
| Enrichment Score (ES) | -0.4769685 |
| Normalized Enrichment Score (NES) | -2.851184 |
| Nominal p-value | 0.0 |
| FDR q-value | 0.0 |
| FWER p-Value | 0.0 |
Table: GSEA Results Summary

  

Fig 1: Enrichment plot: DOROSZKO\_U3054MG\_CLUSTER\_4      
 Profile of the Running ES Score & Positions of GeneSet Members on the Rank Ordered List

  

| PROBE | GENE SYMBOL | GENE\_TITLE | RANK IN GENE LIST | RANK METRIC SCORE | RUNNING ES | CORE ENRICHMENT || 1 | PTTG1 |  |  | 14 | 0.490 | -0.0095 | No |
| 2 | SMS |  |  | 45 | 0.358 | -0.0353 | No |
| 3 | MIF |  |  | 48 | 0.351 | -0.0345 | No |
| 4 | HES6 |  |  | 60 | 0.336 | -0.0423 | No |
| 5 | SRRT |  |  | 74 | 0.314 | -0.0523 | No |
| 6 | HMGB3 |  |  | 80 | 0.311 | -0.0546 | No |
| 7 | BIRC5 |  |  | 130 | 0.271 | -0.0992 | No |
| 8 | NUP205 |  |  | 165 | -0.251 | -0.1297 | No |
| 9 | ZNF519 |  |  | 166 | -0.251 | -0.1277 | No |
| 10 | RFC1 |  |  | 190 | -0.253 | -0.1477 | No |
| 11 | MKI67 |  |  | 206 | -0.254 | -0.1600 | No |
| 12 | DDX11 |  |  | 211 | -0.255 | -0.1618 | No |
| 13 | ERCC6L |  |  | 214 | -0.255 | -0.1617 | No |
| 14 | NAP1L4 |  |  | 215 | -0.255 | -0.1597 | No |
| 15 | FN3KRP |  |  | 218 | -0.255 | -0.1596 | No |
| 16 | PMF1 |  |  | 222 | -0.256 | -0.1605 | No |
| 17 | CNTLN |  |  | 225 | -0.256 | -0.1604 | No |
| 18 | MCM2 |  |  | 226 | -0.256 | -0.1584 | No |
| 19 | PXMP2 |  |  | 233 | -0.257 | -0.1621 | No |
| 20 | NSD2 |  |  | 259 | -0.260 | -0.1839 | No |
| 21 | SASS6 |  |  | 261 | -0.260 | -0.1828 | No |
| 22 | MTHFD1 |  |  | 281 | -0.262 | -0.1989 | No |
| 23 | RBL1 |  |  | 285 | -0.262 | -0.1997 | No |
| 24 | TRIP13 |  |  | 294 | -0.263 | -0.2053 | No |
| 25 | PPP2R3B |  |  | 298 | -0.264 | -0.2060 | No |
| 26 | SDC1 |  |  | 300 | -0.264 | -0.2049 | No |
| 27 | POLA2 |  |  | 312 | -0.264 | -0.2133 | No |
| 28 | HMMR |  |  | 319 | -0.266 | -0.2170 | No |
| 29 | KNTC1 |  |  | 329 | -0.267 | -0.2235 | No |
| 30 | PSMB9 |  |  | 341 | -0.267 | -0.2319 | No |
| 31 | EXOSC8 |  |  | 347 | -0.268 | -0.2345 | No |
| 32 | CTDSPL2 |  |  | 358 | -0.269 | -0.2420 | No |
| 33 | PRR11 |  |  | 361 | -0.269 | -0.2418 | No |
| 34 | CARHSP1 |  |  | 405 | -0.274 | -0.2807 | No |
| 35 | MASTL |  |  | 414 | -0.275 | -0.2861 | No |
| 36 | CEP57 |  |  | 418 | -0.275 | -0.2868 | No |
| 37 | CHCHD3 |  |  | 420 | -0.276 | -0.2856 | No |
| 38 | CSE1L |  |  | 421 | -0.276 | -0.2835 | No |
| 39 | NUP107 |  |  | 422 | -0.276 | -0.2813 | No |
| 40 | UHRF1 |  |  | 438 | -0.278 | -0.2934 | No |
| 41 | TOPBP1 |  |  | 439 | -0.278 | -0.2912 | No |
| 42 | VOPP1 |  |  | 441 | -0.279 | -0.2900 | No |
| 43 | TEDC1 |  |  | 462 | -0.281 | -0.3069 | No |
| 44 | FOXM1 |  |  | 478 | -0.282 | -0.3190 | No |
| 45 | ECT2 |  |  | 481 | -0.283 | -0.3187 | No |
| 46 | PCNT |  |  | 485 | -0.283 | -0.3193 | No |
| 47 | PRADC1 |  |  | 497 | -0.284 | -0.3276 | No |
| 48 | ZGRF1 |  |  | 499 | -0.285 | -0.3263 | No |
| 49 | MCM10 |  |  | 515 | -0.287 | -0.3384 | No |
| 50 | E2F1 |  |  | 521 | -0.287 | -0.3409 | No |
| 51 | CENPP |  |  | 534 | -0.289 | -0.3500 | No |
| 52 | CENPO |  |  | 550 | -0.291 | -0.3621 | No |
| 53 | WDR76 |  |  | 557 | -0.292 | -0.3655 | No |
| 54 | WDHD1 |  |  | 559 | -0.292 | -0.3642 | No |
| 55 | NUP85 |  |  | 562 | -0.292 | -0.3638 | No |
| 56 | NUP155 |  |  | 566 | -0.293 | -0.3643 | No |
| 57 | RFC5 |  |  | 568 | -0.293 | -0.3630 | No |
| 58 | SLBP |  |  | 573 | -0.295 | -0.3645 | No |
| 59 | RFC4 |  |  | 575 | -0.295 | -0.3631 | No |
| 60 | BTG3 |  |  | 581 | -0.296 | -0.3656 | No |
| 61 | SPAG5 |  |  | 605 | -0.301 | -0.3852 | No |
| 62 | RFC2 |  |  | 607 | -0.302 | -0.3837 | No |
| 63 | NCAPH2 |  |  | 616 | -0.303 | -0.3890 | No |
| 64 | MCM3 |  |  | 625 | -0.305 | -0.3942 | No |
| 65 | DCK |  |  | 631 | -0.307 | -0.3966 | No |
| 66 | PRC1 |  |  | 645 | -0.310 | -0.4066 | No |
| 67 | CDK5RAP2 |  |  | 651 | -0.311 | -0.4089 | No |
| 68 | HAUS2 |  |  | 657 | -0.311 | -0.4112 | No |
| 69 | DNMT1 |  |  | 659 | -0.312 | -0.4097 | No |
| 70 | PHGDH |  |  | 662 | -0.312 | -0.4092 | No |
| 71 | HPF1 |  |  | 666 | -0.313 | -0.4096 | No |
| 72 | RAD51C |  |  | 670 | -0.313 | -0.4100 | No |
| 73 | HAUS8 |  |  | 678 | -0.315 | -0.4142 | No |
| 74 | CHAF1A |  |  | 679 | -0.315 | -0.4117 | No |
| 75 | TMPO |  |  | 688 | -0.317 | -0.4169 | No |
| 76 | PLK4 |  |  | 699 | -0.319 | -0.4239 | No |
| 77 | KIF4A |  |  | 706 | -0.320 | -0.4271 | No |
| 78 | MPHOSPH9 |  |  | 713 | -0.321 | -0.4303 | No |
| 79 | FIGNL1 |  |  | 717 | -0.322 | -0.4307 | No |
| 80 | SPC24 |  |  | 747 | -0.330 | -0.4557 | No |
| 81 | WDR34 |  |  | 752 | -0.331 | -0.4569 | No |
| 82 | HADH |  |  | 754 | -0.333 | -0.4553 | No |
| 83 | ASRGL1 |  |  | 760 | -0.333 | -0.4574 | No |
| 84 | PRIM2 |  |  | 764 | -0.336 | -0.4577 | No |
| 85 | SLF1 |  |  | 765 | -0.336 | -0.4550 | No |
| 86 | SAMD1 |  |  | 776 | -0.338 | -0.4619 | No |
| 87 | GINS1 |  |  | 780 | -0.338 | -0.4621 | No |
| 88 | MCM4 |  |  | 783 | -0.339 | -0.4614 | No |
| 89 | PKMYT1 |  |  | 795 | -0.342 | -0.4692 | No |
| 90 | FANCI |  |  | 801 | -0.345 | -0.4712 | No |
| 91 | BRCA2 |  |  | 808 | -0.347 | -0.4742 | Yes |
| 92 | HMGXB4 |  |  | 809 | -0.347 | -0.4715 | Yes |
| 93 | H2AFX |  |  | 810 | -0.347 | -0.4688 | Yes |
| 94 | NCAPD2 |  |  | 811 | -0.347 | -0.4661 | Yes |
| 95 | BLM |  |  | 812 | -0.348 | -0.4633 | Yes |
| 96 | IKBIP |  |  | 813 | -0.348 | -0.4606 | Yes |
| 97 | CENPM |  |  | 818 | -0.349 | -0.4617 | Yes |
| 98 | MAD2L1 |  |  | 819 | -0.350 | -0.4589 | Yes |
| 99 | PHIP |  |  | 823 | -0.350 | -0.4590 | Yes |
| 100 | GINS4 |  |  | 824 | -0.351 | -0.4563 | Yes |
| 101 | BUB3 |  |  | 826 | -0.351 | -0.4545 | Yes |
| 102 | CBX5 |  |  | 842 | -0.356 | -0.4660 | Yes |
| 103 | SHCBP1 |  |  | 844 | -0.356 | -0.4642 | Yes |
| 104 | MRE11 |  |  | 847 | -0.356 | -0.4633 | Yes |
| 105 | ABHD3 |  |  | 848 | -0.357 | -0.4605 | Yes |
| 106 | CENPJ |  |  | 849 | -0.357 | -0.4577 | Yes |
| 107 | CCNE1 |  |  | 850 | -0.357 | -0.4549 | Yes |
| 108 | RFC3 |  |  | 852 | -0.358 | -0.4530 | Yes |
| 109 | ARL6IP6 |  |  | 854 | -0.358 | -0.4512 | Yes |
| 110 | DDIAS |  |  | 859 | -0.359 | -0.4522 | Yes |
| 111 | TFDP1 |  |  | 860 | -0.359 | -0.4493 | Yes |
| 112 | CDCA4 |  |  | 870 | -0.363 | -0.4551 | Yes |
| 113 | OXCT1 |  |  | 879 | -0.366 | -0.4598 | Yes |
| 114 | CCDC15 |  |  | 883 | -0.367 | -0.4598 | Yes |
| 115 | CENPQ |  |  | 884 | -0.367 | -0.4569 | Yes |
| 116 | SMC1A |  |  | 885 | -0.367 | -0.4541 | Yes |
| 117 | STIL |  |  | 888 | -0.369 | -0.4531 | Yes |
| 118 | TCF19 |  |  | 890 | -0.369 | -0.4511 | Yes |
| 119 | PIGX |  |  | 894 | -0.371 | -0.4511 | Yes |
| 120 | KDELC2 |  |  | 897 | -0.372 | -0.4501 | Yes |
| 121 | RAD18 |  |  | 898 | -0.374 | -0.4471 | Yes |
| 122 | CKAP2 |  |  | 900 | -0.375 | -0.4451 | Yes |
| 123 | TK1 |  |  | 902 | -0.375 | -0.4431 | Yes |
| 124 | FANCA |  |  | 910 | -0.378 | -0.4469 | Yes |
| 125 | PARP2 |  |  | 912 | -0.379 | -0.4448 | Yes |
| 126 | TUBG1 |  |  | 917 | -0.381 | -0.4457 | Yes |
| 127 | DSN1 |  |  | 926 | -0.385 | -0.4503 | Yes |
| 128 | MSH2 |  |  | 927 | -0.385 | -0.4472 | Yes |
| 129 | USP1 |  |  | 930 | -0.386 | -0.4461 | Yes |
| 130 | HAT1 |  |  | 933 | -0.387 | -0.4450 | Yes |
| 131 | PSIP1 |  |  | 934 | -0.388 | -0.4419 | Yes |
| 132 | KIF18B |  |  | 935 | -0.388 | -0.4389 | Yes |
| 133 | SLC2A4RG |  |  | 936 | -0.388 | -0.4359 | Yes |
| 134 | DERA |  |  | 937 | -0.389 | -0.4328 | Yes |
| 135 | PPP1R14C |  |  | 939 | -0.390 | -0.4307 | Yes |
| 136 | DIAPH3 |  |  | 943 | -0.390 | -0.4305 | Yes |
| 137 | RPA1 |  |  | 946 | -0.393 | -0.4293 | Yes |
| 138 | TMEM237 |  |  | 953 | -0.396 | -0.4319 | Yes |
| 139 | CDCA7L |  |  | 954 | -0.397 | -0.4288 | Yes |
| 140 | DTL |  |  | 956 | -0.397 | -0.4267 | Yes |
| 141 | PEG10 |  |  | 962 | -0.398 | -0.4283 | Yes |
| 142 | SKA3 |  |  | 965 | -0.399 | -0.4271 | Yes |
| 143 | XPO1 |  |  | 967 | -0.400 | -0.4249 | Yes |
| 144 | HIRIP3 |  |  | 970 | -0.402 | -0.4236 | Yes |
| 145 | RTKN2 |  |  | 973 | -0.403 | -0.4224 | Yes |
| 146 | FANCD2 |  |  | 976 | -0.405 | -0.4211 | Yes |
| 147 | YEATS4 |  |  | 979 | -0.407 | -0.4198 | Yes |
| 148 | FANCL |  |  | 981 | -0.407 | -0.4176 | Yes |
| 149 | CEP78 |  |  | 984 | -0.408 | -0.4163 | Yes |
| 150 | MORC4 |  |  | 987 | -0.408 | -0.4150 | Yes |
| 151 | SMCHD1 |  |  | 990 | -0.411 | -0.4137 | Yes |
| 152 | SPDL1 |  |  | 995 | -0.412 | -0.4142 | Yes |
| 153 | NFATC2IP |  |  | 999 | -0.413 | -0.4139 | Yes |
| 154 | CDC45 |  |  | 1000 | -0.413 | -0.4106 | Yes |
| 155 | POLD3 |  |  | 1002 | -0.415 | -0.4083 | Yes |
| 156 | KIF11 |  |  | 1003 | -0.416 | -0.4051 | Yes |
| 157 | RBBP7 |  |  | 1006 | -0.418 | -0.4037 | Yes |
| 158 | CDC6 |  |  | 1008 | -0.418 | -0.4014 | Yes |
| 159 | MNS1 |  |  | 1009 | -0.419 | -0.3981 | Yes |
| 160 | CCDC34 |  |  | 1013 | -0.421 | -0.3976 | Yes |
| 161 | CYB5A |  |  | 1014 | -0.422 | -0.3943 | Yes |
| 162 | CMC2 |  |  | 1017 | -0.425 | -0.3929 | Yes |
| 163 | MIS18A |  |  | 1020 | -0.427 | -0.3914 | Yes |
| 164 | CENPL |  |  | 1021 | -0.427 | -0.3881 | Yes |
| 165 | TIMELESS |  |  | 1024 | -0.429 | -0.3866 | Yes |
| 166 | SAC3D1 |  |  | 1025 | -0.429 | -0.3832 | Yes |
| 167 | NCAPG2 |  |  | 1027 | -0.430 | -0.3808 | Yes |
| 168 | CCDC14 |  |  | 1030 | -0.433 | -0.3793 | Yes |
| 169 | POLQ |  |  | 1033 | -0.436 | -0.3778 | Yes |
| 170 | TPX2 |  |  | 1034 | -0.437 | -0.3744 | Yes |
| 171 | LIG1 |  |  | 1035 | -0.437 | -0.3709 | Yes |
| 172 | CEP152 |  |  | 1040 | -0.439 | -0.3713 | Yes |
| 173 | FANCB |  |  | 1044 | -0.443 | -0.3707 | Yes |
| 174 | DDB2 |  |  | 1045 | -0.443 | -0.3672 | Yes |
| 175 | KIF22 |  |  | 1051 | -0.446 | -0.3685 | Yes |
| 176 | BRIP1 |  |  | 1052 | -0.447 | -0.3650 | Yes |
| 177 | SMC2 |  |  | 1062 | -0.452 | -0.3700 | Yes |
| 178 | MCM8 |  |  | 1064 | -0.452 | -0.3674 | Yes |
| 179 | CDKN2C |  |  | 1067 | -0.454 | -0.3658 | Yes |
| 180 | EZH2 |  |  | 1070 | -0.455 | -0.3641 | Yes |
| 181 | MMS22L |  |  | 1072 | -0.457 | -0.3615 | Yes |
| 182 | DEPDC1 |  |  | 1074 | -0.457 | -0.3588 | Yes |
| 183 | PSMC3IP |  |  | 1075 | -0.457 | -0.3552 | Yes |
| 184 | MND1 |  |  | 1083 | -0.461 | -0.3583 | Yes |
| 185 | POLA1 |  |  | 1088 | -0.464 | -0.3585 | Yes |
| 186 | ATAD5 |  |  | 1091 | -0.465 | -0.3567 | Yes |
| 187 | NTM |  |  | 1094 | -0.466 | -0.3550 | Yes |
| 188 | ITGB3BP |  |  | 1096 | -0.467 | -0.3523 | Yes |
| 189 | ZWINT |  |  | 1099 | -0.467 | -0.3505 | Yes |
| 190 | BUB1B |  |  | 1111 | -0.477 | -0.3573 | Yes |
| 191 | RACGAP1 |  |  | 1112 | -0.479 | -0.3535 | Yes |
| 192 | MGME1 |  |  | 1113 | -0.479 | -0.3497 | Yes |
| 193 | MCUB |  |  | 1115 | -0.483 | -0.3469 | Yes |
| 194 | E2F8 |  |  | 1116 | -0.485 | -0.3431 | Yes |
| 195 | BARD1 |  |  | 1117 | -0.486 | -0.3393 | Yes |
| 196 | EMP2 |  |  | 1124 | -0.489 | -0.3412 | Yes |
| 197 | RBBP8 |  |  | 1128 | -0.494 | -0.3401 | Yes |
| 198 | NCAPD3 |  |  | 1130 | -0.496 | -0.3372 | Yes |
| 199 | ASF1B |  |  | 1133 | -0.498 | -0.3352 | Yes |
| 200 | OLFM1 |  |  | 1136 | -0.500 | -0.3332 | Yes |
| 201 | PCNA |  |  | 1142 | -0.504 | -0.3340 | Yes |
| 202 | VRK1 |  |  | 1143 | -0.505 | -0.3300 | Yes |
| 203 | IDH2 |  |  | 1146 | -0.508 | -0.3279 | Yes |
| 204 | CKAP2L |  |  | 1147 | -0.508 | -0.3239 | Yes |
| 205 | TMEM106C |  |  | 1148 | -0.509 | -0.3199 | Yes |
| 206 | HSPB11 |  |  | 1149 | -0.511 | -0.3159 | Yes |
| 207 | FAM111B |  |  | 1151 | -0.514 | -0.3129 | Yes |
| 208 | MXD3 |  |  | 1153 | -0.514 | -0.3098 | Yes |
| 209 | NUSAP1 |  |  | 1154 | -0.515 | -0.3057 | Yes |
| 210 | GMNN |  |  | 1155 | -0.516 | -0.3017 | Yes |
| 211 | DBF4 |  |  | 1157 | -0.520 | -0.2985 | Yes |
| 212 | MIS18BP1 |  |  | 1162 | -0.524 | -0.2983 | Yes |
| 213 | CIP2A |  |  | 1165 | -0.533 | -0.2960 | Yes |
| 214 | RRM1 |  |  | 1166 | -0.534 | -0.2918 | Yes |
| 215 | KNL1 |  |  | 1167 | -0.534 | -0.2876 | Yes |
| 216 | KIF14 |  |  | 1168 | -0.535 | -0.2834 | Yes |
| 217 | BRCA1 |  |  | 1171 | -0.541 | -0.2811 | Yes |
| 218 | SGO2 |  |  | 1176 | -0.546 | -0.2806 | Yes |
| 219 | ORC6 |  |  | 1179 | -0.551 | -0.2782 | Yes |
| 220 | XRCC2 |  |  | 1180 | -0.551 | -0.2738 | Yes |
| 221 | CENPU |  |  | 1182 | -0.555 | -0.2704 | Yes |
| 222 | KIF2C |  |  | 1183 | -0.558 | -0.2660 | Yes |
| 223 | CDCA3 |  |  | 1187 | -0.563 | -0.2645 | Yes |
| 224 | ASPM |  |  | 1189 | -0.567 | -0.2610 | Yes |
| 225 | FEN1 |  |  | 1191 | -0.569 | -0.2575 | Yes |
| 226 | NUF2 |  |  | 1194 | -0.574 | -0.2549 | Yes |
| 227 | DHFR |  |  | 1197 | -0.576 | -0.2523 | Yes |
| 228 | CKS1B |  |  | 1198 | -0.577 | -0.2477 | Yes |
| 229 | GTSE1 |  |  | 1210 | -0.602 | -0.2535 | Yes |
| 230 | CLSPN |  |  | 1212 | -0.605 | -0.2497 | Yes |
| 231 | KIF15 |  |  | 1213 | -0.610 | -0.2449 | Yes |
| 232 | CENPK |  |  | 1220 | -0.619 | -0.2458 | Yes |
| 233 | C21orf58 |  |  | 1222 | -0.626 | -0.2418 | Yes |
| 234 | SMC4 |  |  | 1224 | -0.638 | -0.2378 | Yes |
| 235 | MYBL2 |  |  | 1225 | -0.639 | -0.2327 | Yes |
| 236 | CKS2 |  |  | 1226 | -0.641 | -0.2277 | Yes |
| 237 | NCAPH |  |  | 1228 | -0.643 | -0.2236 | Yes |
| 238 | ANLN |  |  | 1229 | -0.646 | -0.2185 | Yes |
| 239 | E2F7 |  |  | 1237 | -0.664 | -0.2200 | Yes |
| 240 | CDCA8 |  |  | 1239 | -0.669 | -0.2157 | Yes |
| 241 | CDCA5 |  |  | 1244 | -0.685 | -0.2141 | Yes |
| 242 | SGO1 |  |  | 1246 | -0.691 | -0.2097 | Yes |
| 243 | AURKB |  |  | 1250 | -0.700 | -0.2070 | Yes |
| 244 | MELK |  |  | 1251 | -0.701 | -0.2015 | Yes |
| 245 | CCNA2 |  |  | 1255 | -0.716 | -0.1988 | Yes |
| 246 | UBE2T |  |  | 1256 | -0.719 | -0.1931 | Yes |
| 247 | ARHGAP11A |  |  | 1262 | -0.735 | -0.1921 | Yes |
| 248 | HMGB2 |  |  | 1263 | -0.736 | -0.1863 | Yes |
| 249 | RAD51AP1 |  |  | 1264 | -0.741 | -0.1805 | Yes |
| 250 | KIF23 |  |  | 1265 | -0.741 | -0.1747 | Yes |
| 251 | KIFC1 |  |  | 1267 | -0.748 | -0.1698 | Yes |
| 252 | TACC3 |  |  | 1268 | -0.751 | -0.1639 | Yes |
| 253 | TYMS |  |  | 1272 | -0.770 | -0.1607 | Yes |
| 254 | CCNE2 |  |  | 1273 | -0.771 | -0.1547 | Yes |
| 255 | ATAD2 |  |  | 1275 | -0.775 | -0.1495 | Yes |
| 256 | NDC80 |  |  | 1278 | -0.810 | -0.1451 | Yes |
| 257 | PCLAF |  |  | 1281 | -0.828 | -0.1405 | Yes |
| 258 | SYNE2 |  |  | 1282 | -0.829 | -0.1340 | Yes |
| 259 | ESCO2 |  |  | 1283 | -0.846 | -0.1273 | Yes |
| 260 | FAM111A |  |  | 1285 | -0.871 | -0.1214 | Yes |
| 261 | HJURP |  |  | 1290 | -0.897 | -0.1182 | Yes |
| 262 | SPC25 |  |  | 1294 | -0.932 | -0.1137 | Yes |
| 263 | RRM2 |  |  | 1295 | -0.933 | -0.1064 | Yes |
| 264 | KPNA2 |  |  | 1297 | -0.972 | -0.0997 | Yes |
| 265 | FBXO5 |  |  | 1301 | -0.992 | -0.0948 | Yes |
| 266 | NCAPG |  |  | 1303 | -1.015 | -0.0878 | Yes |
| 267 | HIST1H1E |  |  | 1309 | -1.319 | -0.0822 | Yes |
| 268 | CDK1 |  |  | 1311 | -1.433 | -0.0719 | Yes |
| 269 | UBE2C |  |  | 1312 | -1.466 | -0.0604 | Yes |
| 270 | TOP2A |  |  | 1313 | -1.528 | -0.0484 | Yes |
| 271 | HIST1H1B |  |  | 1318 | -1.922 | -0.0371 | Yes |
| 272 | HIST1H1D |  |  | 1319 | -1.958 | -0.0217 | Yes |
| 273 | HIST1H4C |  |  | 1320 | -2.769 | -0.0000 | Yes |
Table: GSEA details [plain text format]

  

Fig 2: DOROSZKO\_U3054MG\_CLUSTER\_4: Random ES distribution      
 Gene set null distribution of ES for **DOROSZKO\_U3054MG\_CLUSTER\_4**
